# ABA Sponsors Program on Law and Emerging Infectious Diseases

**DOI:** 10.3201/eid0204.960421

**Published:** 1996

**Authors:** David P. Fidler

**Affiliations:** Indiana University School of Law, Bloomington, Indiana, USA


### ABA Sponsors Program on Law and Emerging Infectious Diseases

In August 1996, the International Health Law Committee, International Law and Practice Section, American Bar Association, sponsored a program entitled "Law and Emerging and Re-Emerging Infectious Diseases" to examine how the emergence and reemergence of infectious diseases affects international and U.S. federal, state, and local law. The issues addressed included international legal rules on infectious disease control and the need for their revision in light of emerging and reemerging infectious diseases; emerging infectious diseases and U.S. federal law, especially as it affects the mission of public health agencies; and the importance of state and local law in dealing with emerging infectious diseases.

Common themes included the challenge posed by emerging infectious diseases as law at every level (international, national, and local) is involved and law in various forms (treaties, constitutions, statutes, and regulations) is affected; the need for legal reform at the international and U.S. federal, state, and local levels; the challenges posed by a complex jurisdictional environment (e.g., World Health Organization's relationship to independent states and U.S. public health agencies' relationships to state governments); in considering legal reform, the need to balance competing policy objectives, such as the control of infectious diseases versus the freedom of global trade and travel or protecting the community versus privacy rights; the interdependence of legal reform efforts in that local and national implementation of revised international rules will be critical to any global strategy; the need to integrate the efforts of lawyers and public health officials to effectively promote epidemiologic principles and objectives; and the massive scope of the emerging diseases threat stemming from not only its global reach but also the long list of causes behind the emergence and reemergence of infectious diseases (e.g., political and medical complacency, international trade, global travel, war, human behavior, environmental degradation, urbanization, poverty, and inadequate public health infrastructures).

The International Health Law Committee program marks a first step in raising awareness in the legal and public health communities of the many and complex legal issues involved in addressing emerging infectious diseases.

For copies of program presentations and other information, contact David P. Fidler by e-mail at davidfidler@law.indiana.edu.

**David P. Fidler**
Indiana University School of Law
Bloomington, Indiana, USA

### A Global Theme Issue: Bibliography of References

Emerging and reemerging infections respect no national boundaries; therefore, they were an appropriate topic for the first "global medical theme issue," introduced in January 1996. This global theme issue was conceived by three editors, Linda Hawes Clever, *The Western Journal of Medicine*, Magne Nylenna, *Journal of the Norwegian Medical Association*, and George D. Lundberg, *Journal of the American Medical Association*, who in 1995 invited the editors of 78 journals worldwide to participate. A year later, 36 journals in 21 countries on six continents published more than 200 articles pertaining to emerging and reemerging global microbial threats (1). The articles addressed topics ranging from factors contributing to increasing antimicrobial resistance to the impact of global warming on infectious disease. While some solutions were suggested, the global issue primarily served as a call to medical communities and people worldwide to identify contributing factors and begin to develop strategies to control emergent infections.

Following this note is a bibliography of articles published by the 36 journals participating in the global theme issue.

**Margaret A. Winker**
Journal of the American Medical Association
Chicago, Illinois, USA

In August 1996, the International Health Law Committee, International Law and Practice Section, American Bar Association, sponsored a program entitled "Law and Emerging and Re-Emerging Infectious Diseases" to examine how the emergence and reemergence of infectious diseases affects international and U.S. federal, state, and local law. The issues addressed included international legal rules on infectious disease control and the need for their revision in light of emerging and reemerging infectious diseases; emerging infectious diseases and U.S. federal law, especially as it affects the mission of public health agencies; and the importance of state and local law in dealing with emerging infectious diseases.

Common themes included the challenge posed by emerging infectious diseases as law at every level (international, national, and local) is involved and law in various forms (treaties, constitutions, statutes, and regulations) is affected; the need for legal reform at the international and U.S. federal, state, and local levels; the challenges posed by a complex jurisdictional environment (e.g., World Health Organization's relationship to independent states and U.S. public health agencies' relationships to state governments); in considering legal reform, the need to balance competing policy objectives, such as the control of infectious diseases versus the freedom of global trade and travel or protecting the community versus privacy rights; the interdependence of legal reform efforts in that local and national implementation of revised international rules will be critical to any global strategy; the need to integrate the efforts of lawyers and public health officials to effectively promote epidemiologic principles and objectives; and the massive scope of the emerging diseases threat stemming from not only its global reach but also the long list of causes behind the emergence and reemergence of infectious diseases (e.g., political and medical complacency, international trade, global travel, war, human behavior, environmental degradation, urbanization, poverty, and inadequate public health infrastructures).

The International Health Law Committee program marks a first step in raising awareness in the legal and public health communities of the many and complex legal issues involved in addressing emerging infectious diseases.

For copies of program presentations and other information, contact David P. Fidler by e-mail at davidfidler@law.indiana.edu.
